# A Plant Kavalactone Desmethoxyyangonin Prevents Inflammation and Fulminant Hepatitis in Mice 

**DOI:** 10.1371/journal.pone.0077626

**Published:** 2013-10-15

**Authors:** Tsui-Wei Chou, Jia-Hua Feng, Chi-Chang Huang, Ya-Wen Cheng, Shih-Chang Chien, Sheng-Yang Wang, Lie-Fen Shyur

**Affiliations:** 1 Department of Culinary Arts, Taoyuan Innovation Institute of Technology, Chungli, Taoyuan County, Taiwan; 2 Agricultural Biotechnology Research Center, Academia Sinica, Taipei, Taiwan; 3 Institute of Plant Biology, National Taiwan University, Taipei, Taiwan; 4 Graduate Institute of Sports Science, National Taiwan Sport University, Guishan Township, Taoyuan County, Taiwan; 5 The Experimental Forest Management Office, National Chung Hsing University, Taichung, Taiwan; 6 Department of Forestry, National Chung Hsing University, Taichung, Taiwan; 7 Graduate Institute of Pharmacognosy, Taipei Medical University, Taipei, Taiwan; Northwestern University Feinberg School of Medicine, United States of America

## Abstract

*Alpinia pricei* Hayata is a Formosan plant which has been popularly used as nutraceutical or folk medicine for inflammation and various disorders. An active compound of the plant rhizomes, desmethoxyyangonin (DMY), was identified in this study for its novel effect against endotoxin lipopolysaccharide (LPS)-stimulated inflammation in murine macrophages and LPS/D-galactosamine (LPS/D-GalN)-induced fulminant hepatitis in mice. DMY was observed to significantly inhibit proliferation and activation of T cells *ex vivo* and the activity of several pro-inflammatory mediators *in vitro*. DMY also protected LPS/D-GalN−induced acute hepatic damages in mice through inhibiting aminotransferases activities and infiltrations of inflammatory macrophages, neutrophils and pathogenic T cells into the liver tissues. In addition, pretreatment with DMY significantly improved the survival rate of LPS/D-GalN−treated mice to 90% (9/10), compared to LPS/D-GalN−treated group (40%, 4/10). UPLC/MS platform-based comparative metabolomics approach was used to explore the serum metabolic profile in fulminant hepatic failure (FHF) mice with or without the DMY pretreatment. The results showed that LPS/D-GalN−induced hepatic damage is likely through perturbing amino acid metabolism, which leads to decreased pyruvate formation via catalysis of aminotransferases, and DMY treatment can prevent to a certain degree of these alterations in metabolic network in mouse caused by LPS/D-GalN. Mechanistic investigation demonstrated that DMY protects LPS or LPS/D-GalN−induced damages in cell or liver tissues mainly through de-regulating IKK/NFκB and Jak2/STAT3 signaling pathways. This report provides evidence-based knowledge to support the rationale for the use of *A. pricei* root extract in anti-inflammation and also its new function as hepatoprotetive agent against fulminant hepatitis.

## Introduction

 Inflammation is a syndrome responsive to pathogen infection or injury. Stimuli−induced production of pro-inflammatory mediators in macrophages, such as TNF-α and NO can cause acute inflammatory responses and may result in inflammatory diseases. LPS from the outer membrane of Gram-negative bacteria promotes secretion of the pro-inflammatory mediators by interacting with the CD14/Toll-like receptor 4/MD^2^ receptor complex in many cell types such as macrophages and endothelial cells [1]. LPS can induce mRNA or protein levels of iNOS, which catalyzes oxidative deamination of L-arginine to produce NO. In the liver, LPS promotes NO production to induce inflammation and liver toxicity by activating iNOS in Kupffer cells, endothelial cells and hepatocytes [2]. As well, some plant natural compounds such as curcumin and tea polyphenols possess hepatoprotective functions by inhibiting iNOS in LPS–induced liver damage [3]. 

 NF-κB plays an important role in regulating LPS−stimulated inflammatory mediators. Activation of NF-κB in response to inflammatory stimuli involves degradation of IκB via cascade activities by phosphorylation of the IKK complex and results in nuclear translocation of NF-κB to bind to the *cis*-element in the promoter regions of various genes such as iNOS and COX-2, thereby inducing their overexpression [4]. The MAPK family members, ERK1/2 and JNK, are signaling molecules that also react to extracellular stimuli and regulate pro-inflammatory cytokine production [5]. Furthermore, STAT3 is involved in inflammatory responses: tyrosine phosphorylation at residue 705 on STAT3 promotes the production of pro-inflammatory cytokines such as IL-6 in LPS−stimulated macrophages [6]. 

 Fulminant hepatic failure (FHF) is associated with severe liver disorders that result in rapid distortion of hepatic function. It is a life-threatening disease, with orthotopic liver transplantation as the only curative treatment at present [7]. Therefore, discovery of effective therapies against FHF is urgently needed. *Alpinia pricei* Hayata (Zingiberaceae) is one of 8 endemic species of *Alpinia* genus in Taiwan. It is commonly found in wild fields and low-attitude mountain areas and frequently used as nutraceuticals or folk medicine for curing flatulence, inflammation, and digestive disturbance. Recent *in vitro* and *in vivo* studies have shown that ethanol extracts of rhizomes of *A. pricei* have bioactivities, such as anticancer [8,9] and improving metabolic syndrome [10]. Chang et al. (2010) identified 3 major compounds, desmethoxyyangonin (DMY), cardamonin, and flavokawain B, from the ethanol extracts of *A. pricei* rhizome and suggested that the extracts had both suppressive and preventive potency against hypercholesterolemia by reducing serum total cholesterol and low-density lipoprotein-cholesterol levels in test animals [11]. 

 In the present study, we showed that DMY inhibited LPS−induced production of NO and iNOS expression in murine RAW 264.7 macrophages. Moreover, DMY effectively prevented LPS/D-GalN–induced acute hepatitis in ICR mice. We used comparative metabolomics with UPLC/Q-TOF mass spectrometry, along with multivariate PLS-DA, to characterize the primary metabolic profile in LPS/D-GalN−challenged mice with or without DMY treatment. This is the first study to elucidate the hepatoprotective activity of DMY and the underlying mechanistic insights.

## Materials and Methods

### Isolation and Structure Elucidation of DMY

**Figure 1:**
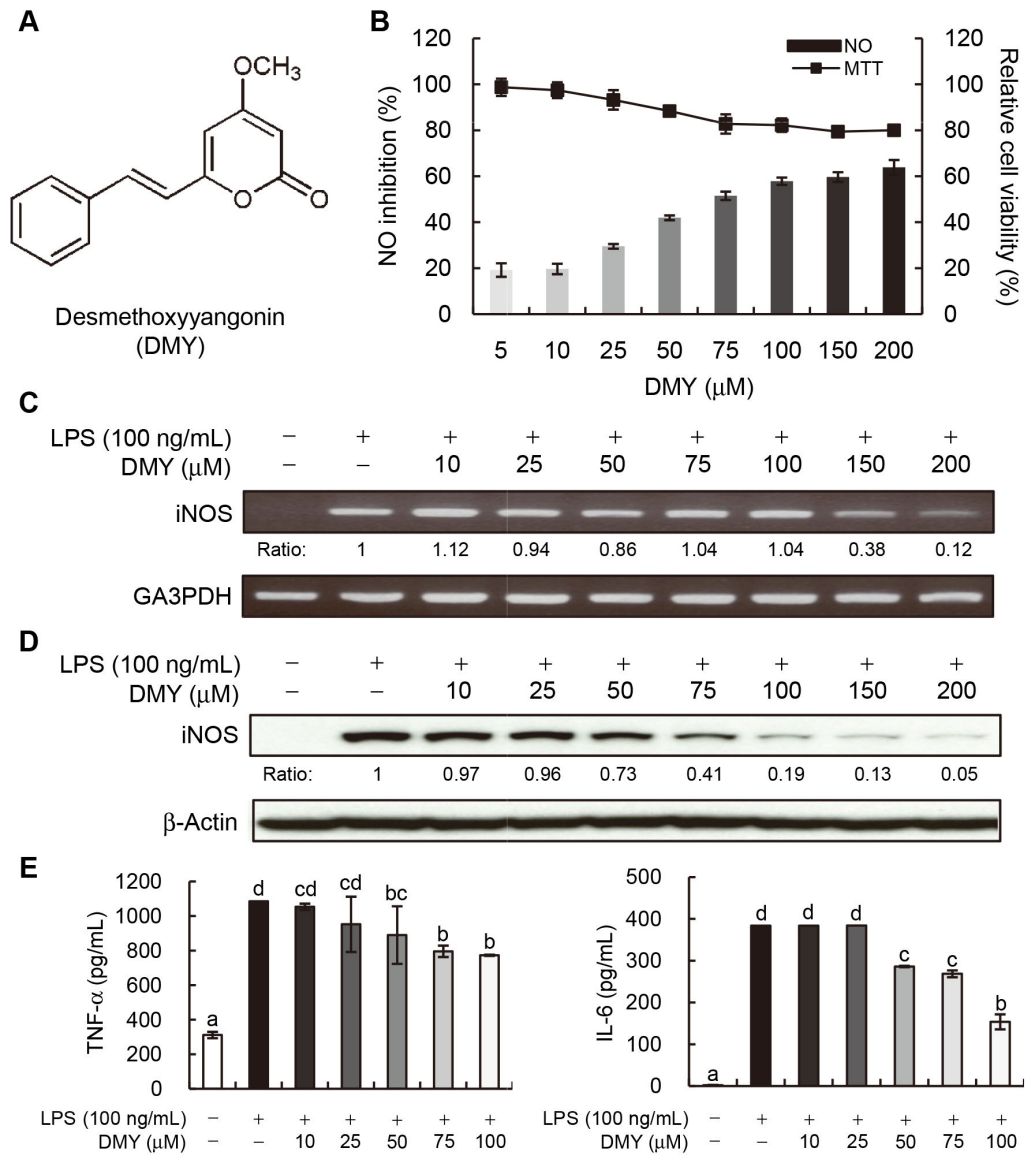
Desmethoxyyangonin (DMY) suppresses pro-inflammatory mediator activity in LPS−stimulated macrophages. (A) Chemical structure of DMY. (B) Inhibition of NO production. Bar graphs: % of NO inhibition; line graphs: relative cell viability (%) by MTT assay. (C) Effect of DMY on iNOS mRNA expression. Cells were treated with the indicated concentrations of DMY for 1 h and then LPS (100 ng/mL) for 6 h. Quantification of iNOS mRNA expression was normalized to GA3PDH by densitometry. Relative ratios are to LPS treatment alone. (D) Effect of DMY on iNOS protein expression. Cells were treated with the indicated concentrations of DMY for 1 h and then LPS (100 ng/mL) for 18 h. Total cellular proteins (30 μg) were resolved by SDS-PAGE and immunoblotted with specific iNOS antibody. Level of iNOS protein was normalized to that of actin, and relative ratios are to LPS treatment alone. (E) Inhibitory effect of DMY on TNF-α and IL-6 secretion. Macrophages (1 × 10^5^ cells/well in 96-well plates) were treated with the indicated concentrations of DMY for 6 h and then stimulated with LPS (100 ng/mL) for 12 h. Data are mean ± SEM, *n* = 3. Means without a common letter differ, *P* < 0.05.

 The roots of *A. pricei* Hayata (Zingiberaceae) were collected from Ping-Tung County located in south Taiwan in March 2007 and were identified by Professor Yen-Hsueh Tseng (National Chung Hsing University). The voucher specimen was deposited in the herbarium of National Museum of Natural Science, Taichung, Taiwan (http://catalog.digitalarchives.tw/item/00/61/e8/e2.html).The air-dried roots (2 kg) were extracted with 10 L 70% ethanol (EtOH) in H_2_O at room temperature. The total crude extract was evaporated to yield the EtOH extract (APE) (166 g). The APE was separated by semi-preparative HPLC. A Luna silica column (250 × 10 mm, Phenomenex Co.) was used with 2 solvent systems, H_2_O (A) and 100% acetonitrile (B). The gradient elution profile was as follows: 0-3 min, 80% A to B; 3-60 min, 80-0% A to B (linear gradient); 60-80 min 0% A to B. The flow rate was 2.5 ml/min and the detector wavelength was set at 280 nm. DMY in the APE was obtained at retention time 32.5 min. The structure of DMY was elucidated by various spectroscopic analyses. UV spectra of test compounds were recorded with use of a Jasco V-550 spectrometer and IR spectra were obtained with a Bio-Rad FTS-40 spectrophotometer. Data from electron-impact MS and high-resolution electron-impact MS were collected with use of a Finnigan MAT-958 mass spectrometer and NMR spectra were recorded with Bruker Avance 500 and 300 MHz FT-NMR spectrometers, at 500 MHz (^1^H) and 75 MHz (^13^C). The structure of DMY (Figure 1A) was elucidated and further confirmed with previously published spectroscopic data [12].

### Chemicals and Reagents

 3-(4,5-Dimethylthiazol-2-yl)-2,5-diphenyl tetrazolium bromide (MTT), D-GalN, LPS, and 4,6-diamidino-2-phenylindole (DAPI) were from Sigma Chemical Co. (St. Louis, MO, USA). DMEM, fetal bovine serum (FBS), and the antibiotic mixture (penicillin-streptomycin) were from Invitrogen (Carlsbad, CA, USA). DMSO, silica gel and RP-18 F_254_ TLC plates were from Merck (Darmstadt, Germany). RP-18 silica gel (75C_18_-OPN) was from Nacalai Tesque (Kyoto, Japan). All other chemicals and solvents were of reagent or HPLC grade.

### Cell Lines and Culture Conditions

 The murine macrophages RAW 264.7 and FL83B hepatocyte cell line were obtained from the American Type Culture Collection (Manassas, VA, USA). Both cell lines were grown in DMEM (Gibco/BRL) supplemented with 10% heat-inactivated FBS, 100 U/mL penicillin, and 100 μg/mL streptomycin, at 37 °C in a humidified 5% CO_2_ incubator.

### Animals

 Male ICR mice were from the Laboratory Animal Center (National Taiwan University, Taipei), given a standard laboratory diet and distilled H_2_O *ad libitum* and kept on a 12 h light/dark cycle at 22 ± 2 °C. All experimental protocols (No: RMiIBASL2010044) were approved by the Institutional Animal Care and Utilization Committee (IACUC), Academia Sinica, Taiwan, R.O.C.

### Measurement of NO Production and Cell Viability

 Marcophages (2 × 10^5^ cells/well in 96-well plate) were treated with the indicated concentrations of DMY for 1 h, then incubated for 24 h with or without 100 ng/μL LPS. Nitrite levels in cell culture medium were determined by the Griess reaction method. Cell viability was examined by MTT assay.

### Reverse Transcriptase PCR (RT-PCR) and Western Blot Analysis

 RT-PCR analysis of iNOS expression and total cellular protein extraction and preparation of cytosolic and nuclear proteins from macrophages were as described [13]. Protein concentration was determined by the Bradford method (Bio-Rad). Protein samples were resolved by 5% to 20% gradient SDS-PAGE and then underwent immunoblotting. Primary antibodies against IκBα, ERK1/2, (JNK1/2, STAT1 and STAT3, α-tubulin, Jak2, and IKK were from Santa Cruz Biotechnology (Santa Cruz, CA, USA), iNOS, PARP, SOCS3, pTyr^701^-STAT1, pTyr^705^-STAT3, phospho-ERK1/2, -JNK1/2, -IκBα, -IKK, and -Jak2 were from Cell Signaling Technology (Danvers, MA, USA) and actin was from Chemicon International (Chemicon-Millipore, Billerica, MA, USA). Appropriate horseradish peroxidase-conjugated secondary antibodies were used. Western blot analysis was performed as described [13]. Protein bands reacting to specific antibody were visualized by use of enhanced chemiluminescence (Amersham) with exposure to chemiluminescence light film (BioMax; Kodak Co.). The resulting images were quantified by densitometry and use of Bio-Profil (Bio-1D 97.04, Vilber-Lourmat).

### Enzymatic Activity Assay of iNOS

Effect of DMY on the enzymatic activity of iNOS enzyme was determined by assessing conversion of substrate L-[^14^C] arginine to product L-[^14^C] citrulline using a NOS activity assay kit from Cayman Chemical (USA).

### Measurement of TNF-α and IL-6 Production in Macrophages

Macrophages (1 × 10^5^ cells/well in 96-well plate) were treated with DMY for 6 h and then LPS (100 ng/mL) for 18~24 h. The concentrations of TNF-α and IL-6 in culture medium were determined by use of ELISA kits from R&D Systems (Minneapolis, MN, USA). 

### [^3^H]Thymidine Incorporation Assay and Determination of IL-2 Level Secreted by CD4^+^ Cells

Splenic CD4^+^ T cells were purified from male ICR mice (4 weeks old) using MACS columns (Miltenyi Biotech) and stimulated with anti-CD3 (0.5 μg/mL) and anti-CD28 (0.5 μg/mL) mAbs in the presence of 0, 25, 50, or 100 μM of DMY, or vehicle alone for 48 h. The IL-2 level secreted in cultural media was determined by the use of an ELISA kit from R&D Systems (Minneapolis, MN, USA). After an 8-h [^3^H]thymidine pulse, the cells were harvested and counted in a PerkinElmer scintillation counter. 

### Effect of DMY on Acute Fulminant Hepatitis in Mice

 The *in vivo* protective effect of DMY on LPS/D-GalN-induced fulminant hepatitis in ICR mice (4 weeks old) was investigated. In total, 24 mice were randomly assigned to 4 groups (*n* = 6 per group) for treatment: vehicle (0.5% DMSO); LPS/D-GalN; 1 mg/kg DMY; and 10 mg/kg DMY. The mice were given intraperitoneally (i.p.) with low and high dose of DMY, respectively for 3 consecutive days and 1 h before injection with 500 ng LPS and 25 mg D-GalN in 250 μL saline. Blood samples were collected by retro-orbital bleeding 8 h after LPS/D-GalN injection, then mice were sacrificed, and serum and liver tissues were collected.

### Measurement of Serum AST and ALT Activities

 Blood samples of test mice were centrifuged at 1400 g at 4 °C for 15 min, and AST and ALT activities in serum supernatants were determined by use of commercial kits from Randox Laboratories (UK).

### Histology and Immunohistochemistry

 Fresh liver tissues were fixed in 10% buffered formalin and then embedded in paraffin. Paraffin-embedded liver samples were sectioned (8 μm) and underwent hematoxylin and eosin (H&E) staining. The formalin-fixed and paraffin-embedded liver sections (4 μm thick) were heat immobilized and deparaffinized with xylene and rehydrated in a graded series of ethanol with a final wash in distilled water. Antigen retrieval involved use of Target Retrieval Solution (DakoCytomation, Glostrup, Denmark) in a Decloaking Chamber (Biocare Medical, Walnut Creek, CA, USA). Sections were stained with F4/80 (pan-macrophage marker), Ly6G (for neutrophils), STAT3, or CD4 mAb and visualized with use of goat anti-rat Cy3-labeled or goat anti-rabbit Cy3-labeled secondary antibodies (Jackson ImmunoResearch). The sections were counterstained with DAPI (1 μg/mL) and visualized at 400× magnification under a fluorescent microscope. *In situ* detection of apoptotic cells involved terminal deoxynucleotidyl transferase-mediated dUTP nick-end labeling (TUNEL) according to the manufacturer’s protocol (Chemicon-Millipore, Billerica, MA, USA). The number of cells positive for F4/80, Ly6G, STAT3, CD4, and TUNEL were analyzed by use of AxioVision (Carl Zeiss MicroImaging, Inc.).

### Survival Study

 Fulminant hepatitis is associated with a high mortality rate; therefore, we studied the protective effect of DMY on LPS/D-GalN−induced mortality in ICR mice. The survival study was performed using a protocol described previously with modifications [14]. A total of 40 mice (4 weeks old) were randomly assigned to four groups (*n* = 10 per group) for treatment with: (1) vehicle (0.5% DMSO); (2) LPS/D-GalN; (3) pretreatment with 1 mg/kg DMY followed by LPS/D-GalN (DMY1+LPS/D-GalN); and (4) pretreatment with 10 mg/kg DMY followed by LPS/D-GalN (DMY10+ LPS/D-GalN). Mice were pretreated *i.p.* with DMY1 and DMY10 for 3 consecutive days and 1 h before LPS/D-GalN administration. The mice were *i.p.* injected with a combination of LPS (200 μg/kg) and D-GalN (800 mg/kg) dissolved in PBS. Mortality was monitored for 48 h after LPS/D-GalN administration.

### Primary Metabolome Analysis of Mice Serum by UPLC/ESI-QTOF MS and Multivariate Analyses

 An LC system (ACQUITY UPLC, Waters, Millford, MA, USA) coupled to a hybrid Q-TOF mass spectrometer was used to analyze the primary metabolome from serum samples of treated mice. The works were carried out in the Metabolomics Core Facility, Academia Sinica, Taiwan. A 10-μL aliquot of sera was applied to a reverse-phase column (HSS T3 C18, 1.8 μm, 2.1 × 150 mm, Waters, Milford, MA, USA), which was kept in a column oven at 40 °C. The mobile phase for positive ion mode consisted of 0.1% formic acid in 2% acetonitrile (buffer A) and 0.1% formic acid in 100% acetonitrile (buffer B). The mobile phase for the negative ion mode consisted of 2% acetonitrile (buffer A) and pure 100% acetonitrile (buffer B). The mobile phase flow rate was 500 μL/min with a 4-min gradient from 0-95% acetonitrile/water. MS involved use of a Waters Micromass Q-TOF micro Synapt High Definition Mass Spectrometer (Synapt HDMS, Waters, Manchester, U.K.) equipped with electrospray ionization in positive and negative modes. The optimal conditions for analysis were source temperature 80 °C, desolvation gas temperature 250 °C, cone gas flow 50 L/h, desolvation gas flow 800 L/h, capillary voltage 2.2 kV for negative mode and 3.0 for positive mode, sampling cone voltage 40.0 V, and extraction cone voltage 4.0 V. A lock mass calibrant of sulfadimethoxine (0.2 μg/mL) in water/acetonitrile (50:50, v/v) was continuously introduced via the second ESI probe (Lock-Spray) at a flow rate of 20-40 μL/min, generating a reference ion for positive ion mode ([M + H]^+^ = 311.0814 and [M + H]^−^ = 309.0658) to ensure accuracy during MS. Data were acquired between m/z 50 and 1000 Da with a 0.2-s scan time and a 0.02-s interscan delay and imported to Markerlynx within Masslynx v4.1 (SCN803, Waters) for peak detection and alignment. The ion intensities for each peak were normalized within each sample, and the 3-D data, peak identifier (RT-m/z pair), sample name, and ion intensity were introduced to software EZinfo v2.0 for principal component analysis (PCA), partial least-squares-discriminant analysis (PLS-DA) and orthogonal projection to latent structures (OPLS) analysis.

### Statistical Analysis

 Data are expressed as mean ± SEM. Statistical analyses were conducted using a SAS program (SAS Institute), and significance of differences between treatments was determined by ANOVA. *P* < 0.05 was considered statistically significant.

## Results

### DMY Suppresses LPS–induced NO Production and iNOS Expression in RAW 264.7 Cells

 The *in vitro* LPS−stimulated inflammation in RAW264.7 cell system was used to evaluate the effect of DMY (Figure 1A) on NO production. By determination of nitrite level (equivalent to NO level) in RAW 264.7 culture medium 24 h after LPS stimulation, DMY dose-dependently inhibited NO production with IC_50_ value 70 μM (Figure 1B). The IC_50_ values for inhibiting NO production in LPS-induced macrophages for a LPS antagonist resatorvid and a well-known anti-inflammatory phytocompound curcumin, used as reference controls in this study, were of 300 nM and 18 μM, respectively. Moreover, DMY showed little cytotoxicity to macrophages at a high concentration, 150~200 μM, because 80% of cells were viable, as examined by MTT assay (Figure 1B). DMY at the same concentrations had no cytotoxic effects on murine FL83B hepatocytes (data not shown). 

 iNOS catalyzes the oxidative deamination of arginine to produce NO. We elucidated whether the NO inhibition effect of DMY was due to its regulation of iNOS gene and protein expression. The results showed that 62% to 88% of iNOS mRNA expression was inhibited by 150~200 μM DMY as compared with LPS alone (Figure 1C), and the iNOS protein level was more significantly inhibited by DMY in a dose-dependent manner, with more than 60% of the protein level of iNOS inhibited at > 75 μM DMY as compared with LPS alone (Figure 1D). In parallel, we performed an iNOS enzymatic assay to examine whether the inhibition of NO production by DMY is also through direct inhibition of iNOS enzyme activity. Our result showed that the iNOS enzyme activity was not changed by incubation with 200 μM of DMY as 94.0% and 94.5% of substrate L-[^14^C] arginine were converted to product L-[^14^C] citrulline in the presence or absence of compound DMY, respectively. Therefore, it was concluded that DMY suppresses NO production in LPS–stimulated macrophages by down-regulating iNOS gene and protein expression.

### DMY Suppresses LPS–induced Proinflammatory Mediators in RAW 264.7 Cells

 LPS−activated macrophages release proinflammatory cytokines such as TNF-α and IL-6, which could cause inflammatory disorders, sepsis or other liver injuries [15]. Thus, we measured secreted TNF-α and IL-6 in the culture medium of LPS–stimulated RAW 264.7 cells with or without DMY treatment. The secretion of TNF-α and IL-6 was decreased with increasing concentration of DMY. DMY at 50~100 μM could significantly inhibit the secretion of both TNF-α and IL-6 in LPS–stimulated macrophages (Figure 1E). 

### DMY Suppresses Phosphorylated STAT3 Expression and Attenuates LPS-induced IL-6 Production in Macrophages

**Figure 2:**
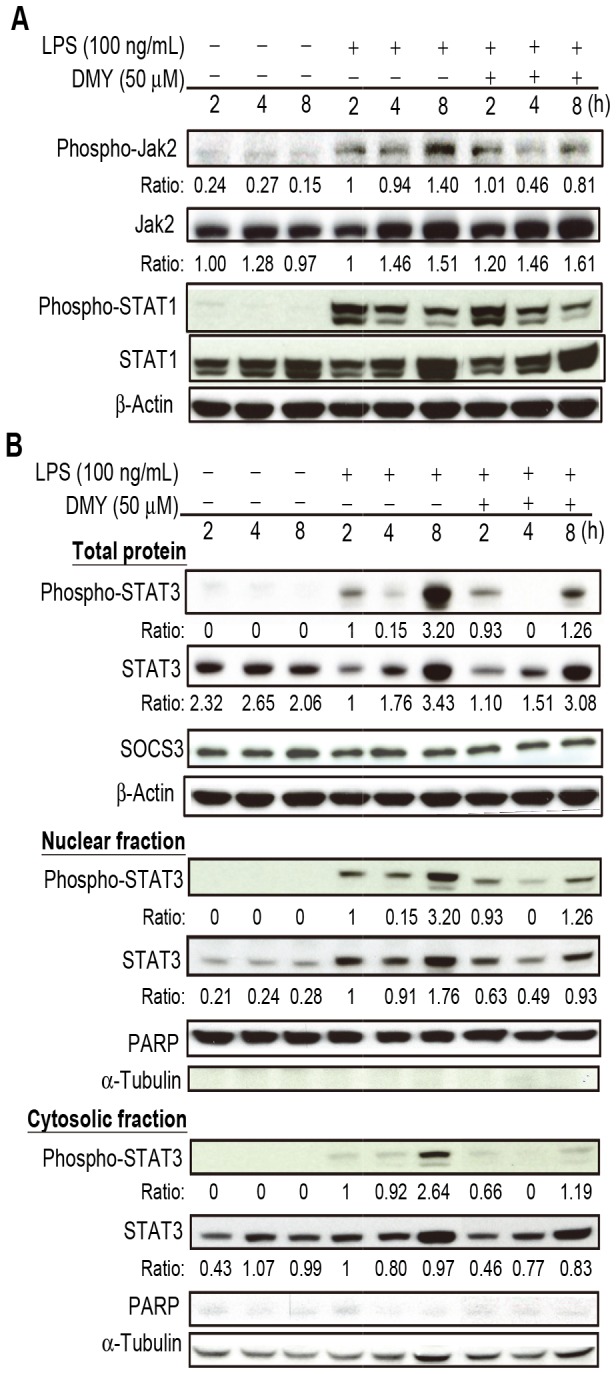
The effect of DMY treatment on Jak/STAT signaling pathway in LPS−stimulated macrophages. Cells were pre-treated with 50 μM DMY for 1 h, then with LPS treatment for 2, 4, and 8 h. Western blot analysis of (A) Phospho- and total Jak2 and STAT1 levels, (B) Total STAT3, phospho-STAT3, and SOCS3 levels and the levels of STAT3 and phospho-STAT3 in nuclear and cytosolic fractions. PARP and α-tubulin were used as internal control of nuclear and cytosolic proteins, respectively.

 STAT1 and STAT3 are phosphorylated due to activation of Jak/STAT signaling in LPS−treated macrophages [16]. The tyrosine phosphorylation of STAT3 promotes the release of pro-inflammatory cytokines such as IL-6 [6]. We investigated whether the inhibitory effect of DMY on IL-6 release was associated with activation of STAT3 and its upstream Jak kinase in LPS−stimulated RAW 264.7 cells. The level of Jak2 and its phosphorylated form was significantly increased with LPS stimulation (Figure 2A). Treatment with 50 μM DMY significantly suppressed phospho-Jak2 but not total Jak2 protein level (Figure 2A). DMY had no effect on the LPS−induced activation of STAT1 in macrophages, as shown by the level of phosphorylated STAT1 (pTyr^701^-STAT1) protein (Figure 2A). SOCS3 plays a role in negative regulation of IL-6 signaling [14] but was not responsive to DMY treatment (Figure 2B). Because nuclear translocation of STAT3 occurs after Jak2 kinase activation [16], we analyzed nuclear and cytosolic fractions of pTyr^705^-STAT3 and STAT3 in addition to their total protein expression. PARP and α-tubulin were used as internal and loading controls for nuclear and cytosolic protein fractions, respectively. DMY significantly suppressed LPS−induced increasing of STAT3 and phospho-STAT3 levels in the total proteins and in both nuclear and cytosolic fractions, as compared with control treatment at 8 h (Figure 2B). These data suggest that DMY suppression of LPS−induced pro-inflammatory cytokine production may be mediated in part by the Jak2/STAT3 signaling pathway. 

### DMY Inhibits LPS−activated IKK Signaling Pathway in Macrophages

**Figure 3:**
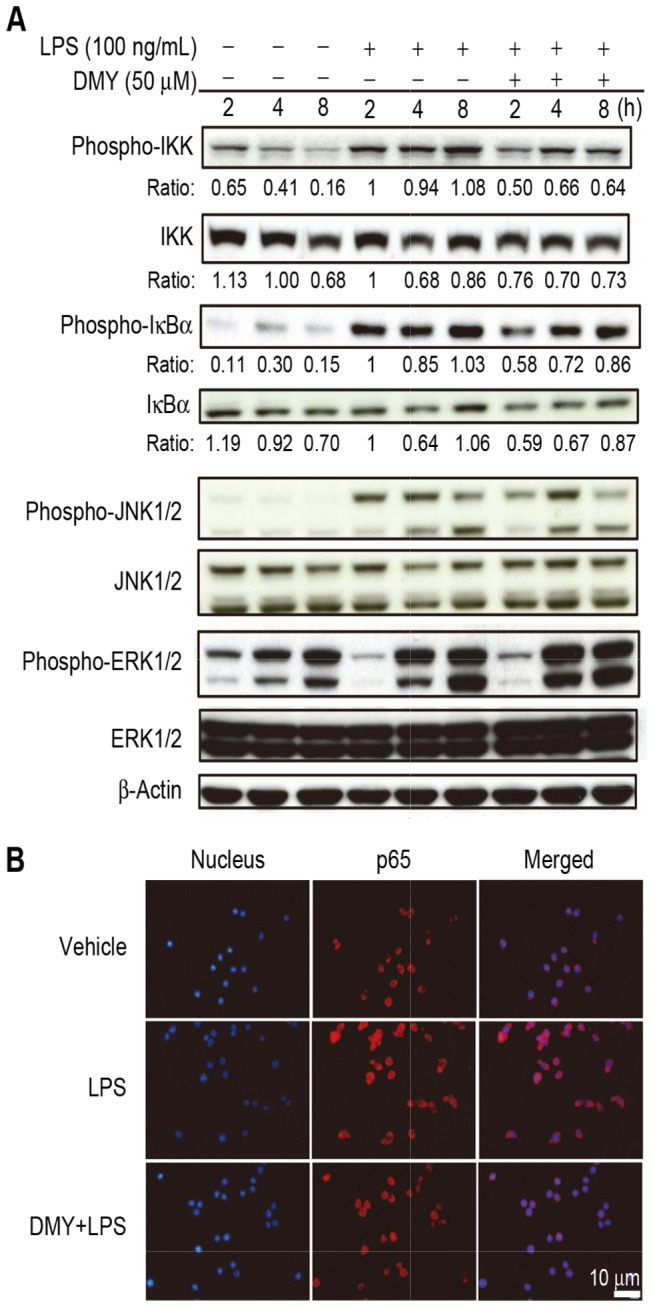
The effect of DMY treatment on NF-κB and MAPK signaling pathway in LPS−stimulated macrophages. Cells were pre-treated with 50 μM DMY for 1 h, then with LPS treatment for 2, 4, and 8 h. Western blot analysis of (A) Phospho- and total IKK, IκBα, JNK1/2 and ERK1/2 levels. (B) Immunofluorescence staining of nuclear translocation of NF-κB(p65) protein. Cells were stained with DAPI (nuclear marker, blue) and rabbit anti-p65 antibody (red).

 Because iNOS gene expression can be regulated by the transcription factor NF-κB, we investigated whether DMY could affect the activation of IKK and IκBα to regulate nuclear translocation of NF-κB in macrophages. The level of phosphorylated forms of IKK and IκBα protein increased with LPS treatment, which were both suppressed with DMY pretreatment (Figure 3A). The IKK and IKBα total protein levels were also lower in DMY–treated cells than cells with LPS stimulation only. Furthermore, DMY inhibited the LPS−induced translocation of NF-κB from the cytosol to nucleus in macrophages (Figure 3B). These data indicate that activation of IKK with LPS stimulation further activates IκBα for its ubiquitination and proteasomal degradation to promote NF-κB nuclear translocation were inhibited by DMY. JNK 1/2 and ERK 1/2, involved in MAPK pathway, have been shown to play a role in LPS−induced inflammation in macrophages. We found no difference in protein levels of JNK1/2 and ERK1/2 or their phosphorylated forms in LPS−stimulated cells with or without DMY treatment (Figure 3A), so DMY had no effect on the LPS−activated MAPK pathway in macrophages. 

### Effect of DMY on LPS/D-GalN−induced Fulminant Hepatitis in ICR Mice

**Figure 4:**
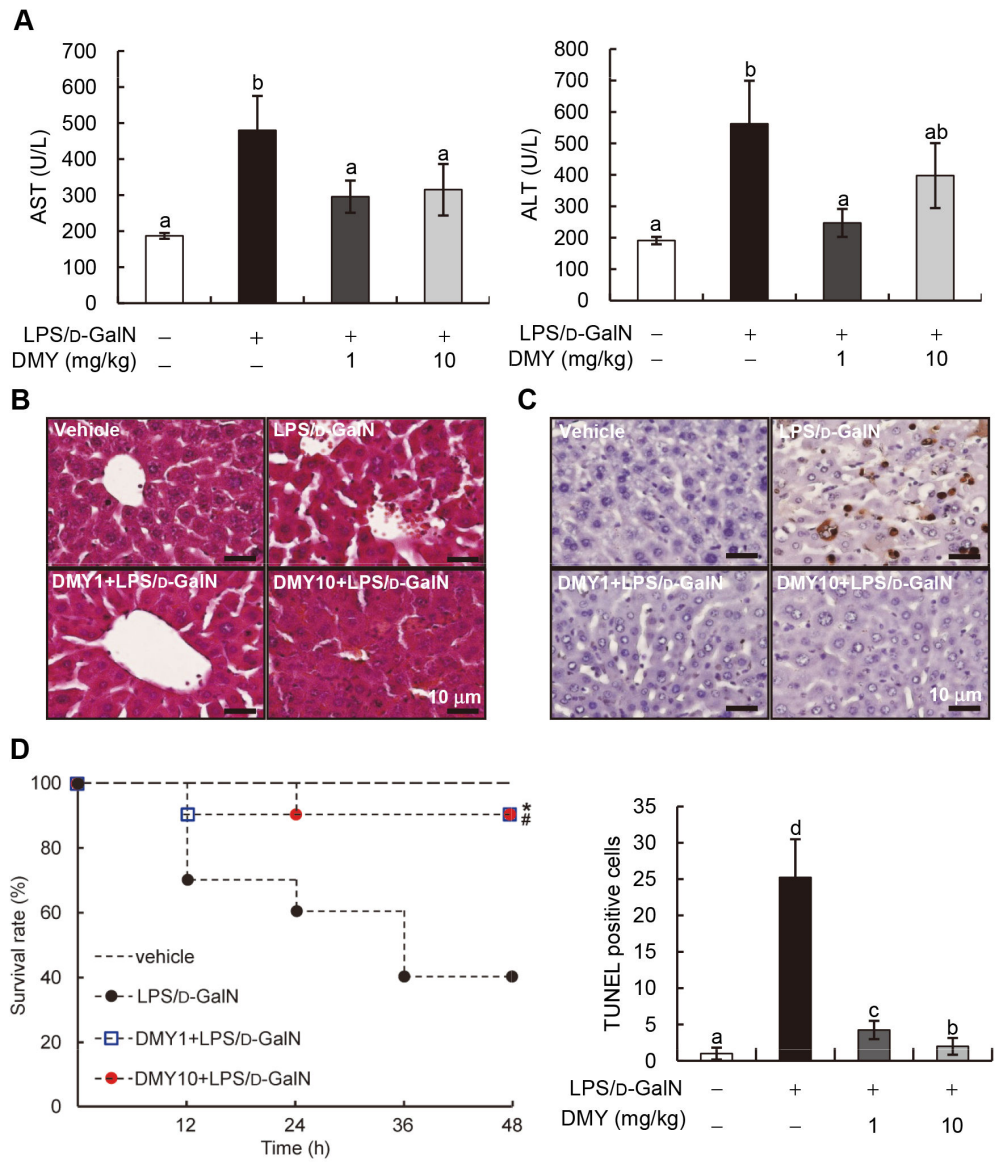
Protective effects of DMY on LPS/D-GalN−induced acute liver dysfunction in mice. Mice were pretreated with DMY (1 and 10 mg/kg) for three consecutive days, then LPS/D-GalN for 8 h. (A) Serum levels of AST and ALT with or without treatment. Data are mean ± SEM, *n* = 6. Means without a common letter differ, *P* < 0.05. (B) Hematoxylin and eosin staining of mouse livers. (C) TUNEL assay of apoptosis in mouse liver with LPS/D-GalN challenge or DMY treatment. Representative image of each treatment group is shown. Brownish cells are TUNEL-positive apoptotic cells. (D) Survival rate of LPS/D-GalN-challenged mice with or without DMY-pretreatment. ^*,#^ *P* < 0.05, significant differences within treatment groups and the LPS/D-GalN group (log rank test).

 Because DMY had specific activity in inhibiting LPS−induced inflammation in macrophages *in vitro*, we next examined whether DMY could effectively prevent LPS/D-GalN–induced fulminant hepatitis in mice. Serum AST and ALT activities have been used clinically as indicators of hepatic injury [17]. Hence, we first measured serum AST and ALT levels to evaluate LPS/D-GalN–induced liver injury in mice. Serum AST and ALT levels were increased (~2.7-fold) in LPS/D-GalN–challenged mouse serum as compared with control serum (Figure 4A). Pretreatment with 1 or 10 mg/kg DMY significantly suppressed (1.7- to 2.4-fold decrease) LPS/D-GalN–induced increase in AST and ALT activity in mouse serum (*P* < 0.05) (Figure 4A). No statistical difference for the preventive effect of 1 and 10 mg/kg DMY treatments was observed. As compared with control mouse liver, LPS/D-GalN–challenged liver showed loss of sinusoidal cells and erythrocyte influx (hemorrhage) as compared with controls, which was attenuated by pretreatment with DMY (Figure 4B). Furthermore, DMY significantly decreased the number of apoptotic cells (TUNEL positive-stained cells) in the liver tissues of LPS/D-GalN–challenged mice (Figure 4C). Thus, DMY could effectively protect the mouse liver against LPS/D-GalN–induced damage. 

### DMY Pretreatment Prevents LPS/D-GalN–induced Macrophage and Neutrophil Recruitment and STAT3 Expression in Mouse Liver

**Figure 5:**
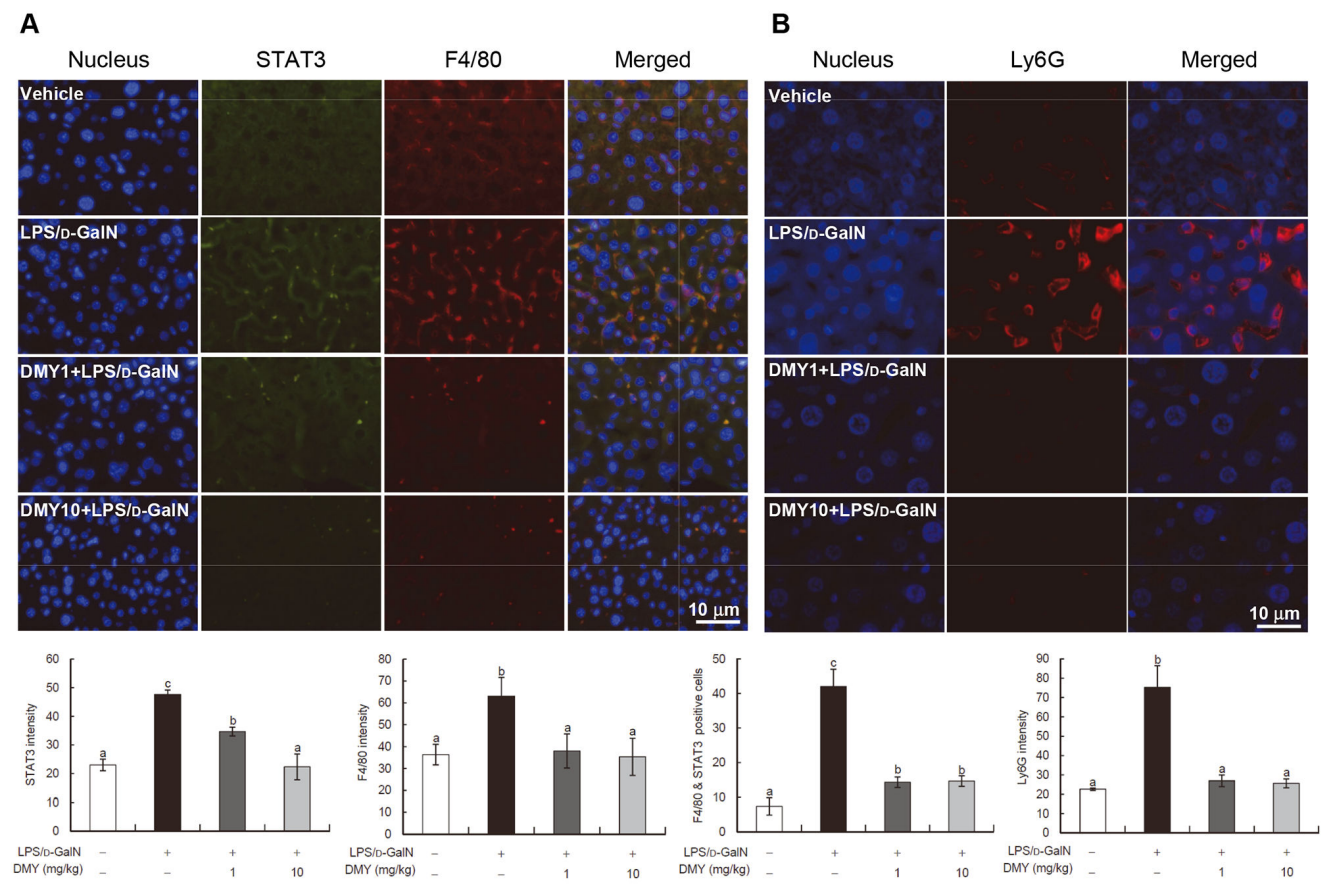
Immunohistochemistry of liver tissues from LPS/D-GalN–challenged mice with or without DMY pretreatment. Immunofluorescence staining and quantification of DMY inhibiting STAT3 and F4/80 (macrophage) infiltration (A) and Ly6G (neutrophil) infiltration (B) in LPS/D-GalN−treated mouse liver. Data are mean ± SEM, *n* = 4. Means without a common letter differ, *P* < 0.05.

 Immunohistochemical analysis showed that macrophages (F4/80-stained cells) and neutrophils (Ly6G-stained cells) infiltrated into liver tissues with LPS/D-GalN challenge, and DMY pretreatment could significantly prevent the infiltration (Figure 5A, B). As well, LPS/D-GalN–increased STAT3 protein expression was significantly reduced with DMY treatment (*P* < 0.05) (Figure 5A). Furthermore, on the basis of the quantification results from the dual staining of F4/80 and STAT3 (F4/80 & STAT3 positive cells), it is suggested that most of the STAT3 proteins were originated from the infiltrated macrophages. These data are in good agreement with the *in vitro* inhibitory effect of DMY on STAT3 protein level in macrophages with LPS challenge.

### DMY Inhibits CD4^+^ Cell Activation and Proliferation, and Prevents Pathogenic CD4^+^ Cell Infiltration into Liver Tissues of Mice Treated with LPS/D-GalN

**Figure 6:**
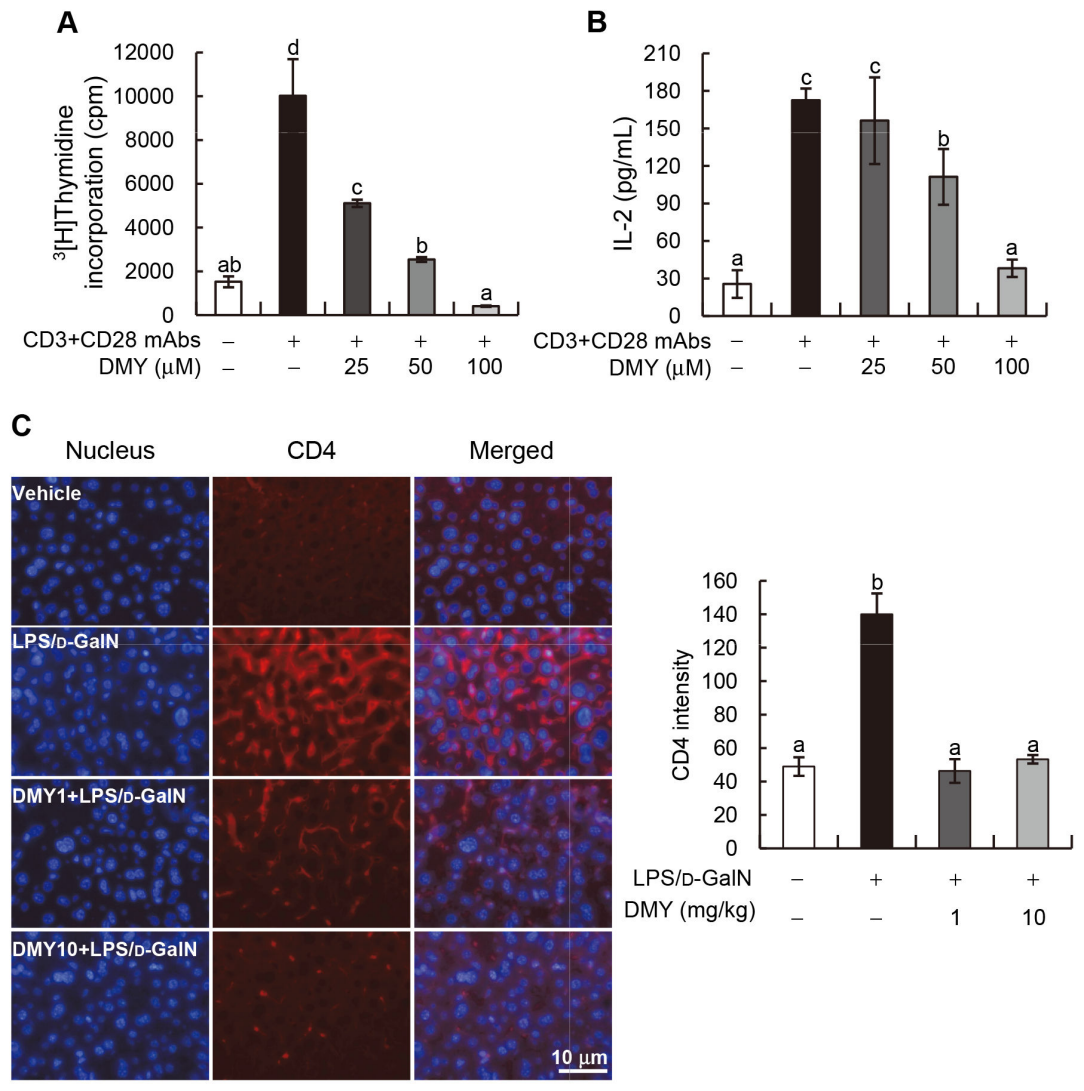
DMY inhibits T cells proliferation *ex* *vivo* and T cells infiltration into mouse liver tissues. (A) Splenic CD4^+^ T cells were purified from male ICR mouse and stimulated with vehicle alone or anti-CD3/anti-CD28 mAbs plus 0, 25, 50, or 100 μM of DMY for 48 h. [^3^H]Thymidine incorporation in T cells was determined. (B) The concentration of IL-2 in cultural media of T cells was determined by ELISA. (C) Immunohistochemistry analysis of mouse liver tissues against CD4 mAb. Data are mean ± SEM, *n* = 3. Means without a common letter differ, *P* < 0.05.

In LPS/D-GalN−induced fulminant hepatitis, T cells play a pivotal role in production of various cytokines that lead to liver injury [18]. We, therefore, investigated whether DMY treatment can inhibit T cell activation and proliferation, and/or prevent T cell infiltration into the liver tissue of LPS/D-GalN−treated mice. Splenic CD4^+^ cells were purified and activated with anti-CD3 and anti-CD28 mAbs with or without co-incubation with DMY at 25, 50, and 100 μM, respectively. The results of [^3^H]Thymidine incorporation and the level of secreted IL-2 in CD4^+^ cells indicated that DMY can dose-dependently inhibit CD4^+^ cell activation and proliferation (*P* < 0.05; Figure 6A, B). Immunohistochemical analysis further showed that the pathogenic T cells significantly infiltrated into LPS/D-GalN−treated mouse livers, which were inhibited by DMY treatment (Figure 6C). These results suggest that the T cell activities related to the onset of fulminant hepatitis can be modulated by DMY treatment.

### DMY Protects Mice Against LPS/D-GalN−induced Mortality

 Our data have shown that DMY has a hepatoprotective effect against LPS/D-GalN−induced fulminant hepatitis in mice. We further examined whether DMY treatment protects against LPS/D-GalN−induced mortality in mice. The data in Figure 4D show that only 40% (4/10) of animals survived after 48 h following LPS/D-GalN insult compared to the vehicle group. Both pretreatment with DMY1 and DMY10 significantly improved the survival rate of LPS/D-GalN−treated mice to 90% (9/10), with *P* = 0.0257 and *P* = 0.0217, respectively, compared to LPS/D-GalN−treated group. This result indicates that DMY treatment can protect mice from LPS/D-GalN−induced mortality.

### Comparative Metabolomic Analysis of Mouse Serum

**Figure 7:**
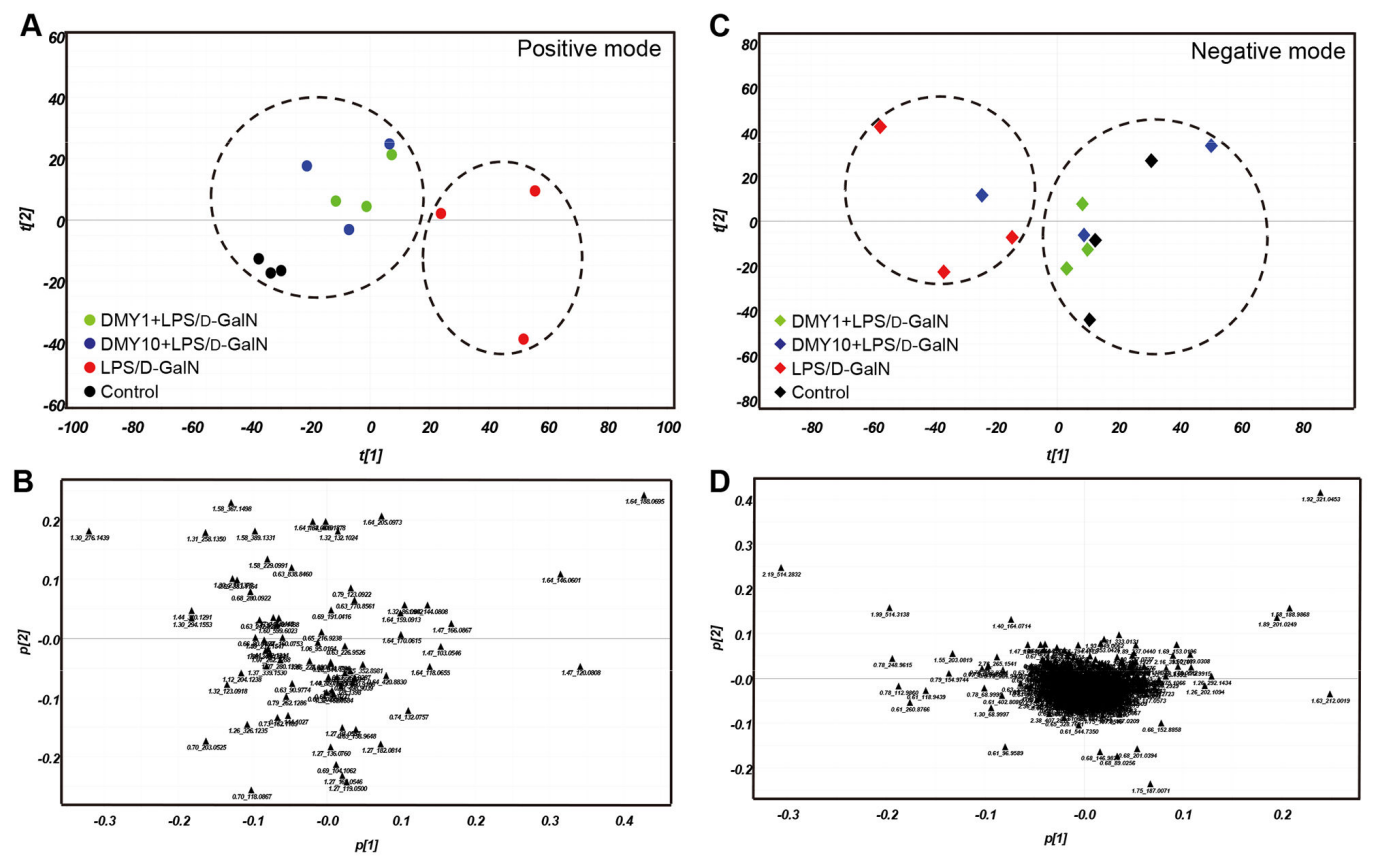
Comparative metabolomic analysis of mouse serum. Score plots (A, C) and corresponding loading plots (B, D) for PCA of UPLC/QTOF MS data from mice treated with vehicle control, LPS/D-GalN, and LPS/D-GalN–challenged mice pretreated with DMY1 and DMY10 (*n* = 3 in each group). *Dashed* *circles* group pairs of samples from LPS/D-GalN–challenged group vs. other treatment groups. The ions most responsible for the variance of the score plots are indicated by their distance from the origin. The metabolites are labeled according their retention times in the chromatogram and *m*/*z* values.

 We used UPLC/ESI-QTOF MS coupled with multiple pattern recognition methods such as PCA and PLS-DA to phenotype the serum metabolome of test mice. Sera from 3 mice from each treatment group -- vehicle control, LPS/D-GalN challenge, and pretreatment with both DMY doses before LPS/D-GalN challenge -- underwent global metabolic profiling in both ESI positive and negative modes. PCA results can provide an overview of all observations or samples in a dataset. Each point in a score plot represents the mass data in an individual test mouse, and groupings, trends, and outliers can be observed. The score plots show a similar metabolome when data points cluster together or closer and when they disperse, which suggests compositionally different metabolomes. The score plots from analysis of positive- and negative-mode ions (Figure 7A, C) showed 2 clusters: LPS/D-GalN–challenged mice and vehicle and DMY-pretreated mice (except an outlier of 10 mg/kg DMY treatment in negative ion mode), which suggests that LPS/D-GalN altered the metabolic fluctuations that could be prevented by DMY treatment in mice. The corresponding loading plots for PCA of UPLC/QTOF MS data present the impact of each metabolite on this PCA clustering (Figure 7B, D). The distance of an ion from the origin represents its contribution to the clustering of different groups on the PCA. 

### Metabolite Profiles and Variations in Mouse Serum with Different Treatments

 We identified 29 metabolites from the spectral dataset and verified them by reference standards (Table 1). From 15 amino acids identified, the total amino acid concentration was significantly higher with LPS/D-GalN challenge than the vehicle control and DMY pretreatment (*P* < 0.05). Ile/Leu, Phe, and Trp were significantly increased in level and Pro was decreased in level in LPS/D-GalN–challenged mouse sera. The 1- and 10-mg/kg DMY (DMY1 and DMY10) pretreatment could attenuate the increase in Ile/Leu, Phe, or Trp levels or decrease in Pro level. As well, the concentrations of gluconeogenic amino acids, branched-chain amino acids, and aromatic amino acids were greater with LPS/D-GalN challenge than vehicle treatment (*P* < 0.05) and were all lowered by DMY pretreatment. Furthermore, as compared with vehicle treatment, LPS/D-GalN challenge decreased the levels of pyruvate, uric acid, myoinositol, and the reduced form of glutathione (GSH) in mouse serum. With DMY1 and DMY10 plus LPS/D-GalN, the levels of pyruvate, myoinositol and GSH were increased (*P* < 0.05) as compared with LPS/D-GalN alone, which suggests that DMY can prevent the LPS/D-GalN–altered metabolism of amino acids and other metabolites involved in glycolysis in mice. However, DMY could not prevent the LPS/D-GalN–decreased uric acid level in mouse serum and could further decrease nicotinamide content, the product of NAD^+^ and NADP^+^, in mice. LPS/D-GalN challenge may cause some disturbances in fatty acid metabolism, as seen in level of dodecanoic acid, palmitic acid, and oleic acid, and some differences in data occurred with DMY treatment, but the variations were too great to make any suggestions. 

**Table 1:** Metabolites identified in the serum of mice treated with vehicle control and LPS/D-GalN, as well as LPS/D-GalN–challenged mice pretreated with 1 and 10 mg/kg desmethoxyyangonin (DMY1 and DMY10), respectively.

Metabolites	Vehicle control	LPS/D-GalN challenged	DMY1+LPS/D-GalN	DMY10+LPS/D-GalN
Alanine	1.06 ± 0.07^a^	0.86 ± 0.16^a^	1.01 ± 0.20^a^	0.96 ± 0.21^a^
Arginine	1.39 ± 0.09^a^	1.28 ± 0.36^a^	1.45 ± 0.37^a^	1.31 ± 0.24^a^
Aspartate	0.72 ± 0.09^a^	0.68 ± 0.31^a^	0.81 ± 0.31^a^	0.65 ± 0.26^a^
Glutamate	3.02 ± 0.65^a^	2.13 ± 0.79^a^	2.82 ± 0.51^a^	2.53 ± 0.42^a^
Glutamine	3.97 ± 0.18^a^	4.72 ± 1.16^ab^	5.74 ± 0.82^b^	5.51 ± 0.86^ab^
Glycine	8.87 ± 1.85^a^	8.21 ± 1.22^a^	13.62 ± 2.68^b^	8.64 ± 2.13^a^
Histidine	2.24 ± 0.60^a^	3.21 ± 1.33^a^	2.78 ± 0.28^a^	2.19 ± 0.40^a^
Isoleucine/Leucine	115.02 ± 20.36^a^	150.73 ± 19.03^b.^	122.61 ± 17.92^ab^	127.64 ± 4.68^ab^
Methionine	12.28 ± 1.81^a^	11.02 ± 1.86 ^a^	11.58 ± 4.21^a^	11.31 ± 1.34^a^
Phenylalanine	82.76 ± 7.66^a^	107.85 ± 6.73^b^	96.97 ± 8.73^b^	88.45 ± 5.44^ab^
Proline	1.38 ± 0.04^a^	1.17 ± 0.14^b^	1.40 ± 0.06^a^	1.10 ± 0.08^b^
Serine	0.58 ± 0.05^a^	0.51 ± 0.10^a^	0.66 ± 0.06^a^	0.59 ± 0.20^a^
Tryptophan	122.15 ± 7.46^a^	180.83 ± 36.05^b^	154.26 ± 12.66^ab^	162.31 ± 13.19^b^
Valine	11.07 ± 2.23^a^	15.03 ± 3.31^a^	12.35 ± 2.24^a^	13.03 ± 0.12^a^
**Total amino acids**	366.52 ± 35.22^a^	488.23 ± 70.22^b^	428.23 ± 70.22^ab^	426.22 ± 15.80^ab^
**GAA**	225.43 ± 27.26^a^	289.75 ± 31.05^b^	255.17 ± 20.68^ab^	246.57 ± 6.80^ab^
**BCAA**	126.09 ± 22.56^a^	165.76 ± 22.20^b^	134.95 ± 20.02^ab^	140.66 ± 4.68^ab^
**AAA**	204.90 ± 11.05^a^	288.68 ± 42.43^b^	251.23 ± 21.27^ab^	250.76 ± 15.09^ab^
Taurine (-)	5.58 ± 1.50^a^	5.80 ± 2.23^a^	4.96 ± 1.13^a^	4.15 ± 0.88^a^
Glutathione reduced	0.22 ± 0.06^a^	0.08 ± 0.02^b^	0.11 ± 0.06^a^	0.14 ± 0.03^a^
Pyruvate	2.29 ± 0.39^ab^	1.44 ± 0.41^b^	3.17 ± 1.16^a^	3.03 ± 0.22^a^
L-lactate	68.53 ± 20.98^a^	46.14 ± 11.79^a^	70.51 ± 22.93^a^	61.34 ± 6.11^a^
Myoinositol	30.59 ± 5.14^a^	21.24 ± 2.80^b^	23.62 ± 3.23^ab^	27.15 ± 3.82^ab^
Nicotinamide	2.15 ± 0.44^a^	1.88 ± 0.18^ab^	1.41 ± 0.06^c^	1.50 ± 0.06^bc^
Creatine	2.56 ± 0.39^a^	2.59 ± 0.18^a^	2.63 ± 0.32^a^	2.43 ± 0.44^a^
Uric acid	2.39 ± 0.15^a^	1.81 ± 0.51^b^	1.36 ± 0.11^b^	1.57 ± 0.28^b^
Carnitine	3.21 ± 0.72^a^	2.35 ± 0.75^a^	2.71 ± 0.40^a^	2.44 ± 0.60^a^
Dodecanoic acid	343.08 ± 23.71^a^	646.62 ± 562.93^a^	418.09 ± 110.56^a^	684.23 ± 204.50^a^
Hippuric acid	11.11 ± 3.49^a^	10.70 ± 5.14^a^	11.45 ± 3.52^a^	10.72 ± 6.80^a^
Palmitic acid	6484.4 ± 737.74^a^	5396.3 ± 1930.9^ab^	5835.5 ± 768.71^ab^	3829.3 ± 994.73^b^
Stearic acid	1436.0 ± 697.54^a^	1277.0 ± 200.27^a^	1571.0 ± 81.51^a^	828.15 ± 289.16^a^
Oleic acid	11887.3 ± 1546.5^a^	8605.8 ± 3955.5^a^	10393.4 ± 2843.3^a^	6638.0 ± 2348.5^a^
Oleamide	1.17 ± 0.30^ab^	0.96 ± 0.48^a^	1.35 ± 0.13^ab^	1.61 ± 0.27^b^

Note: Data are means ± SEM, *n* = 5. (*P* < 0.05, ANOVA). GAA (gluconeogenic amino acids): aspartate, serine, glycine, glutamine, threonine, alanine, proline, tyrosine, phenylalanine, valine, leucine, and isoleucine. BCAA (branched chain amino acids): leucine, isoleucine and valine. AAA (aromatic amino acids): phenylalanine and tryptophan.

## Discussion

 LPS/D-GalN–induced acute liver injury in mice has been widely used as an experimental animal model for FHF because it is similar to the mechanism of FHF observed clinically [19]. In this model, D-GalN can potentiate the acute toxicity of LPS, whereby LPS activates macrophages and kupffer cells to produce TNF-α, then induce hepatocyte apoptosis in the early stage of LPS/D-GalN–induced hepatitis in mice [20]. Here, we showed that pretreatment with 1 or 10 mg/kg DMY significantly suppressed LPS/D-GalN–induced increase in AST and ALT levels in mouse serum and increased apoptotic cells in mouse liver. Furthermore, DMY pretreatment prevented the infiltration of macrophages and neutrophils, inflammatory cell populations, and pathogenic T cells into liver lobules of mice. DMY also effectively inhibited LPS−stimulated TNF-α secretion in macrophages and IL-2 secretion in activated primary CD4^+^ T cell. We conclude here that DMY protects animals against fuminant hepatitis induced by LPS/D-GalN at least in part by attenuating inflammatory cell and CD4^+^ T cell activities. 

 Hepatic inflammation is responsible for liver cell damage, fibrosis and cirrhosis, and LPS plays a critical role in FHF in humans. In the present report, we used LPS−induced inflammatory responses and cascades in the macrophage cell system to elucidate the anti-inflammation and hepatoprotective activities and the underlying mechanisms of DMY. DMY suppressed LPS−induced iNOS expression in RAW 264.7 cells, which inhibited NO production in the cells. Moreover, DMY suppressed LPS−induced TNF-α and IL-6 secretion in RAW 264.7 cells. Complex intracellular signaling networks, such as NF-κB and MAPK pathways are involved in regulation of pro-inflammatory mediator production in response to a broad range of stimuli, including ischemia/reperfusion, ultraviolet light, and microbial infection [5]. The translocation of NF-κB to the nucleus depends on the phosphorylation, ubiquitination and proteolytic degradation of IκB. Nuclear NF-κB can transactivate several inflammation or immune-related genes such as iNOS, COX-2, and TNF-α [4]. We found that DMY treatment could decrease the level of phospho-IKK and phospho-IκBα protein in LPS−treated cells, as well as prevent nuclear translocation of NF-κB. These data suggest that DMY inhibition of iNOS expression at protein and translational levels and NO production are likely via deregulating an NF-κB-related signaling pathway. We have observed that DMY treatment did not alter the expression of COX-2 gene or protein in LPS−stimulated macrophages (data not shown). Although MAPKs can transmit environmental stimuli into the nucleus to regulate the activity of cytokines or other inflammatory mediators such as TNF-α, IL-6 and NO, in this study, DMY treatment did not have a discernable effect on LPS−induced activation of JNK and ERK proteins, as revealed by no changes in levels of phospho-JNK and phospho-ERK protein in macrophages.

Besides NF-κB and MAPK pathways, STAT signaling has a role in inflammatory responses. STAT signaling is activated by upstream JAK kinase via phosphorylation of the tyrosine residue on STAT1 or STAT3, then dimerization and nuclear translocation of STAT protein to activate its responsive gene(s) [16]. Both STAT1 and 3 have an important role in LPS−stimulated inflammatory responses in macrophages: STAT1 induces iNOS gene expression and promotes NO production and STAT3 promotes secretion of pro-inflammatory cytokines such as IL-6 [21,22]. In our study, DMY treatment inhibited the level of phospho-Jak2 and -STAT3 in LPS−induced macrophages but did not affect phospho-STAT1 level. Moreover, DMY−prevented nuclear translocation of STAT3 was likely via inhibiting STAT3 expression because its protein level in the cytosolic fraction was decreased with DMY treatment, which could lead to lower levels of STAT3 and pTyr^705^-STAT3 detected in nucleus. In addition, DMY effectively reduced STAT3 protein expression in LPS/D-GalN−treated mouse liver, so both *in vitro* and *in vivo* findings of DMY deregulating STAT3 expression are in good agreement. Nevertheless, the deregulation of STAT3 protein and its activation by DMY may lead to inhibiting IL-6 expression. Our results demonstrate that the anti-inflammatory and hepatoprotective effects of DMY are by modulating NF-κB and Jak2/STAT3 signaling pathways.

**Figure 8:**
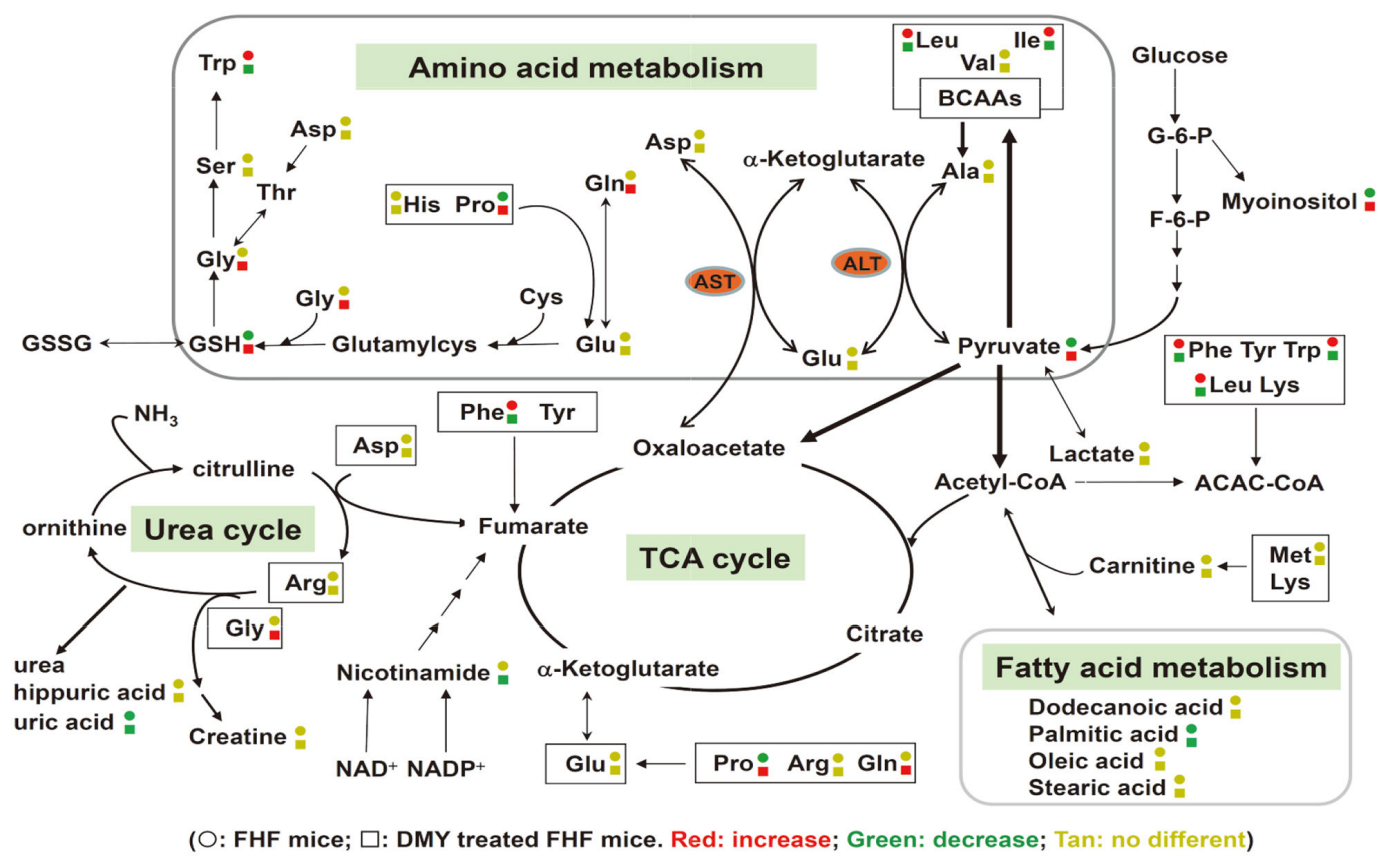
Working hypothesis of the metabolic mechanisms of LPS/D-GalN– induced hepatic toxicity and protection by DMY. Symbol circle and square represent the relative metabolite changes in the LPS/D-GalN−challenged group and the DMY treatment group, respectively. The decrease, increase and no difference in levels with statistical significance are presented in green, red and tan, respectively.

 Metabolomics is an emerging technology for analyzing global or target metabolic profiles to monitor disease development, drug metabolism, and chemical toxicology [23]. It has gained importance in biotechnology applications such as the diagnosis and phenotyping of plants, evaluating genetically modified crops, and analyzing the improvement of compositional quality of crops [24]. It is also useful in taxonomic classification and understanding the pharmacological activity of herbal medicines [25,26]. We used a UPLC/MS platform-based comparative metabolomics approach to explore the serum metabolic profile in FHF mice with or without the phytoagent DMY pretreatment. The metabolic perturbation in multiple pathways with LPS/D-GalN challenge and DMY treatment are summarized (Figure 8). The total amino acid concentrations of 15 amino acids were higher in mice with FHF than controls and DMY treated groups, a similar pattern to the concentrations of gluconeogenic amino acids (GAA), branched-chain (BCAA) and aromatic amino acids (AAA). Epidemiological study revealed that patients with FHF typically show elevated AAA, BCAA and a wide range of other AAs [27]; in addition, an important clinical parameter in FHF is decreased BCAA/AAA ratio [28]. We observed these phenomena in FHF mouse serum, which was also consistently observed in plasma in mice with fulminant hepatitis as analyzed by GC/TOF MS [29]. DMY pretreatment could lower the elevated level of total or different groups of AAs in FHF mice by mainly decreasing the levels of Ile/Leu, Phe, and Trp. The level of taurine, a sulfur-containing AA produced by oxidation of cysteine, was elevated in alcohol-treated 129S *Ppara*-null mice with the occurrence of high reactive oxygen species (ROS) generated [30]. We found no difference in taurin level in control, DMY-treated, or FHF mice. However, the antioxidant GSH, a tripeptide formed by amino acids Glu, Cys and Gly, which can prevent cellular damage caused by ROS, was significantly decreased in FHF mice as compared with controls; notably, DMY pretreatment could prevent some degree of attenuating formation of GSH in FHF mice. These data suggest an effect of DMY on anti-oxidative stress in animals. 

Normally, blood Ala transports to liver via glucose-alanine cycle to generate pyruvate, which is a source of carbon atoms for gluconeogenesis. However, pyruvate can form alanine through transamination by ALT and is also the substrate for forming BCAAs (Figure 8). In some cases of severe hepatic toxicity, such as cirrhosis, BCAAs are used in muscle to create alanine, which is then shuttled to the liver for facilitating pyruvate formation. Previously, Ala level was found to be largely decreased in liver tissue of rats with CCl_4_-induced acute hepatic injury [31]. We observed an increase in BCAAs, with no change in Ala level, a decrease in pyruvate level, and elevated ALT activity in FHF mouse serum, which suggests that ALT is involved in affecting the dynamics of BCAA and pyruvate contents in the mouse. Therefore, LPS/D-GalN−induced hepatic damage is likely through perturbing amino acid metabolism, which leads to decreased pyruvate formation via catalysis of aminotransferases, in turn, attenuating the glycolysis pathway through the TCA cycle for energy production in animals with FHF. These alterations in metabolic network caused by LPS/D-GalN in ICR mouse can be prevented to a certain degree by DMY treatment. 

Previous studies used GC/TOF MS to analyze the changes in metabolites and metabolic pathways in the plasma of BALB/c mice with FHF induced by LPS/D-GalN, with plasma levels of phosphate, glucose, lactate, and β-hydroxybutyrate suggested to be potential markers of FHF diagnosis and prognosis [29]. We observed there was no significant change in the lactate level in both treated and untreated animals, and myoinositol formed from glucose was found decreased in FHF mouse serum, but increased in that of mice with DMY treatment. We did not detect significant levels of metabolites phosphate, glucose, and β-hydroxybutyrate in our system, which may be due to different mouse strains (BALB/c vs. ICR mice) and treatment variables, body fluid tested (plasma vs. serum), and analytical techniques (GC/MS vs. LC/MS). 

We conclude that LPS/D-GalN–induced FHF in animals may reflect damage in liver function and changes in whole-body metabolism. Investigation of metabolic changes and metabolic pathways may provide a rationale for developing nutritional support or alternative options of therapy for FHF. This study provides evidence-based health benefits of the active compound DMY from rhizome of nutraceutical plant *A. pricei* for anti-inflammation and acute hepatitis.
